# Investigation of the occupancy ratio dependence for microlens arrays on diamond

**DOI:** 10.1039/c8ra03803a

**Published:** 2018-08-20

**Authors:** Tian-Fei Zhu, Jiao Fu, Zongchen Liu, Yan Liang, Wei Wang, Feng Wen, Jingwen Zhang, Hong-Xing Wang

**Affiliations:** Institute of Wide Band Gap Semiconductors, Xi'an Jiaotong University Xi'an 710049 PR China hxwangcn@mail.xjtu.edu.cn

## Abstract

Diamond microlens arrays with a high occupancy ratio were fabricated by an improved thermal reflow method. Our resultes show that the occupancy ratio of a photoresist mask could be improved with optimizing the reflow temperature, time and photoresist thickness during the reflow process. The fabricated microlens arrays exhibited a uniform arrangement and good optical performance.

## Abstract 2

Diamond microlens arrays with a high occupancy ratio were fabricated by an improved thermal reflow method.

## Graphical abstract

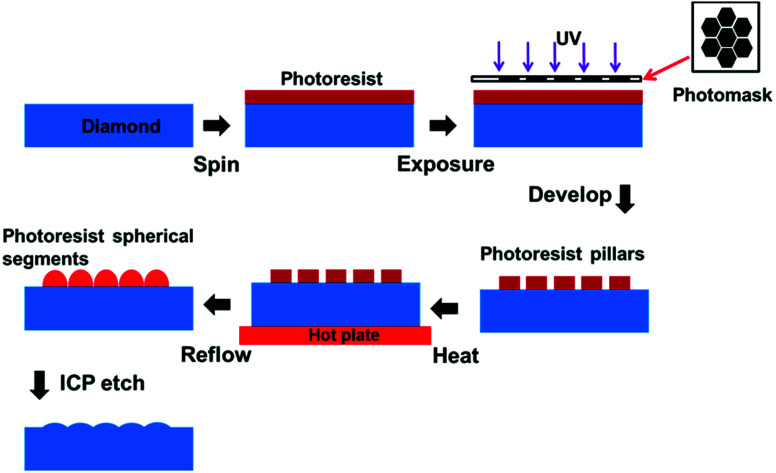

Microlenses have many advantages in optics and optoelectronic fields.^1–5^ They can improve the imaging resolution in imaging fibres^6^ and extend the field of view of a camera.^7^ In addition, microlenses can improve the light extraction efficiency in LED devices^8,9^ and the incidence efficiency in solar cell photovoltaic and photodetector devices.^3,10^ With the progress of new technology, some devices need to work in harsh conditions, such as high temperature or strong acid atmospheres, and materials with high performance are expected. So far, materials such as sapphire,^9^ SiC^11^ and GaN^12^ have been applied for microlenses. In contrast with such materials, diamond has many outstanding properties, such as high hardness, stable chemical inertness, high thermal conductivity and wide transmittance window, making it advantageous for use in many optical and optoelectronic applications, such as lasers and photodetectors.^3,13,14^ Microlens structures on a diamond device are desired to achieve improved performance. For instance, a larger photo-incident area is formed with a microlens structure to achieve an improved photocurrent in a diamond photodetector.^3^ Furthermore, a homogenizer with compact microlens arrays (MLAs) is utilized to achieve a well-distributed laser beam.^15^ In these devices, the occupancy ratio of the MLAs is important for enhancing their optical performance. To date, although much attention has been paid to achieving diamond microlenses with specific parameters, such as a controllable or large focal length and height,^5,16–18^ few research studies have been focused on the fabrication of diamond MLAs with a high fill factor. In this work, compact MLAs on diamond were fabricated. The dependencies of the MLAs' occupancy ratio on the thermal reflow temperature, time and pillar thickness were then investigated. It was found that the fabricated MLAs exhibited good imaging performance and a well-distributed size in a projection experiment.

The substrates used in the present work were double-sided, polished, chemical vapour deposited (CVD), (001) single crystal diamonds with dimensions of 3 × 3 × 0.5 mm^3^. The results of the diamond MLAs fabrication processes are schematically illustrated in Fig. 1. SPR 220-7.0 and 220-3.0 photoresists (PRs) were used. The PR was spun on the diamond substrate at 3500 rpm to 8000 rpm, resulting in a PR thickness of 3.0 to 7.0 μm. Then, the standard photolithography process was used to form hexagonal PR pillars with a circumcircle diameter of about 80 μm. The space between two neighbouring pillars was about 5 μm. By holding the sample on a hot plate for various times at a temperature above 120 °C, the pillars melted and turned into spherical segments.

**Fig. 1:**
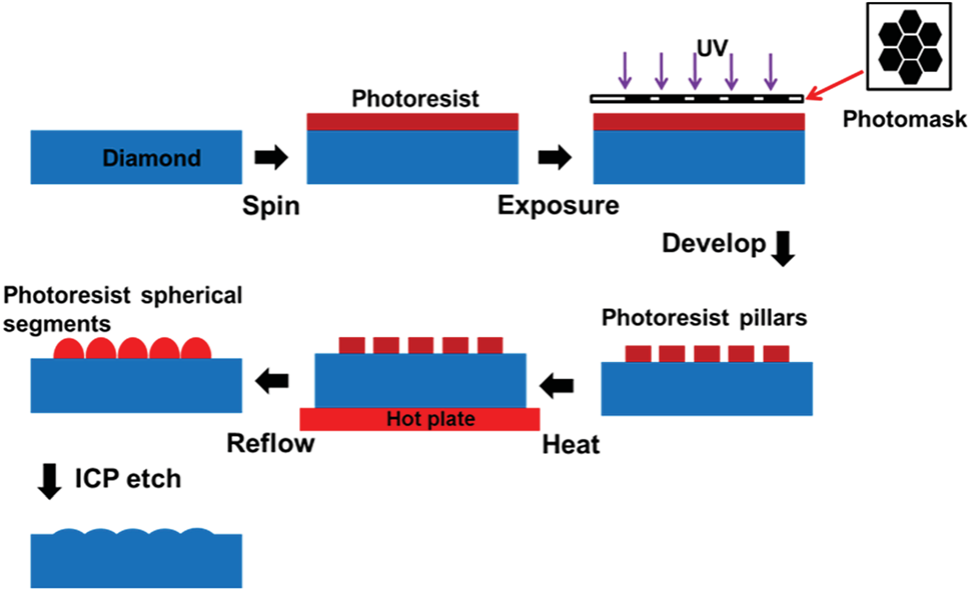
Schematic of the diamond MLAs fabrication process.

Finally, the PR patterns obtained with the optimized experiment parameters were transferred onto the diamond surface using an inductively coupled plasma (ICP) etch process with O_2_ and Ar as the etching gas. The flow rates of O_2_ and Ar were 40 and 15 sccm, respectively. The chamber pressure, coil power and bias voltage were 10 mTorr, 450 W and −120 V, respectively.

The PR mask patterns were investigated by optical microscopy (OM) and laser confocal scanning microscopy (LCSM). The morphologies of the diamond MLAs were characterized by OM and atomic force microscopy (AFM). The optical performances of the diamond MLAs were investigated using a modified optical system.

To investigate the factors that affect the occupancy ratio of the MLAs, a series of experiments focused on the reflow temperature, reflow time and PR pillar thickness dependencies of the PR spherical segment arrays' occupancy ratio were carried out. The results, shown in Fig. 2, suggest that the PR arrays occupancy ratio is dependent on the reflow temperature, reflow time and PR pillar thickness.

**Fig. 2:**
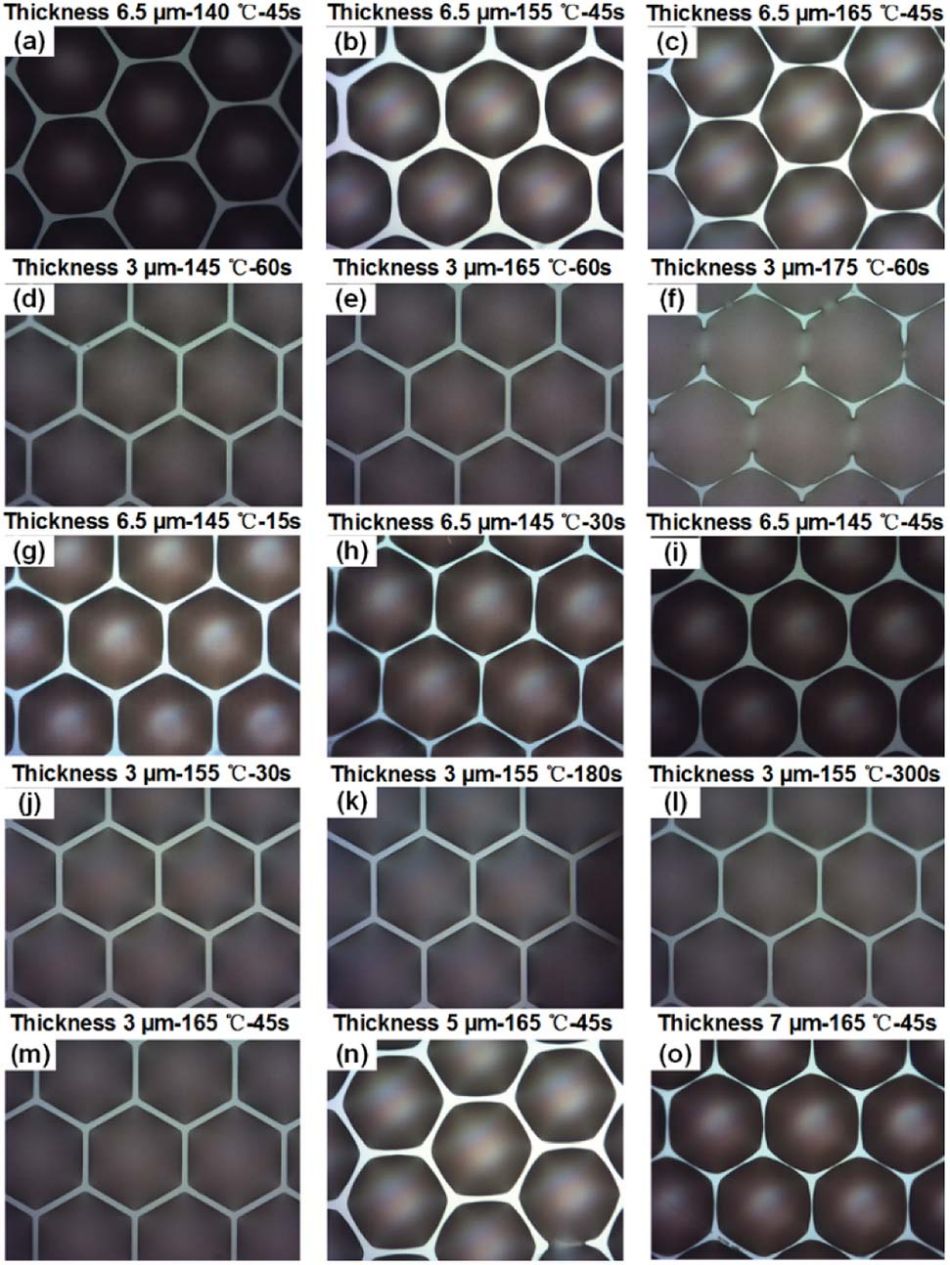
(a–c) Pillars with a thickness of 6.5 μm after thermal reflow treatment for 45 s; (d–f) pillars with a thickness of 3.0 μm after thermal reflow treatment for 60 s; (g–i) pillars with a thickness of 6.5 μm after thermal reflow treatment at 145 °C for 15 s, 30 s and 45 s; (j–l) pillars with a thickness of 3.0 μm after thermal reflow treatment at 155 °C for 30 s, 180 s and 300 s; (m–o) pillars with a thickness of 3.0, 5.0 and 7.0 μm after thermal reflow treatment at 165 °C for 45 s.

PR pillars with a thickness of 6.5 and 3.0 μm were thermally reflowed at various temperatures for 45 s and 60 s, respectively. All the pillars changed to spherical segments, as shown in Fig. 2(a)–(f). For the 6.5 μm case, these pillars were reflowed at 140 °C, 155 °C and 165 °C, respectively. As shown in Fig. 2(a), the contact line shape of the PR spherical segment remained hexagonal after thermal treatment at 140 °C. As the temperature increased, the contact line shape changed to a circle, and expanded to a little larger than that of the pillars, as shown in Fig. 2(b) and (c). This suggests that the occupancy ratio of the spherical segment arrays is improved with increasing temperature. However, for the 3.0 μm case, when the reflowing temperature reached 165 °C, the PR spherical segment still had a hexagonal contact line. When the temperature was further increased to 175 °C, a connected pattern appeared, suggesting an over-heated contact line of the PR spherical segment.

Also, PR pillars with a thickness of 6.5 and 3.0 μm were thermally reflowed for various times at a temperature of 145 °C and 155 °C, respectively. For the 6.5 μm pillars, as shown in Fig. 2(g)–(i), the contact line of the reflowed PR spherical segment is enlarged with increasing the thermal reflow time at a temperature of 145 °C, indicating a controllable occupancy ratio of the spherical segment arrays. In addition, the contact line shape of the PR spherical segment is changed to circle with increasing reflow time. For the 3.0 μm case, the contact line evolution was not similar to the 6.5 μm case. As shown in Fig. 2(j)–(l), with the initial 3.0 μm height pillars, the contact line shape remained unchanged with increasing reflow time at a temperature of 155 °C.

To gain an insight in to the dependence of the PR spherical segment contact line on the pillar thickness, pillars with three different thicknesses were thermally reflowed at a temperature of 165 °C for 45 s. As shown in Fig. 2(m)–(o), the contact line is enlarged with an increased thickness at the same temperature, suggesting a dependence of the contact line on the pillar thickness. With the increasing contact line of the PR patterns, the occupancy ratio of the spherical segment arrays is improved. This shows that a high occupancy ratio of spherical segment is relatively easy to obtain with a thick PR pillar.

The spherical segment height determines the curvature of microlens when the base diameter is fixed. Hence, the reflow temperature, time and pillar thickness dependencies of the spherical segment heights were investigated. From Fig. 3, the height of the spherical segment obtained from both thick and thin pillars was almost independent of the reflow temperature and time. Meanwhile, the height was dependent on the initial pillar thickness, suggesting a thickness-based curvature controllability. In our case, the base diameter was regarded as constant since enlargement of the contact line was too small to affect the curvature. Besides, the about 10% variation of the photoresist spherical segment is thought to have originated from a change in the photoresist film thickness caused by the photoresist coating process.

**Fig. 3:**
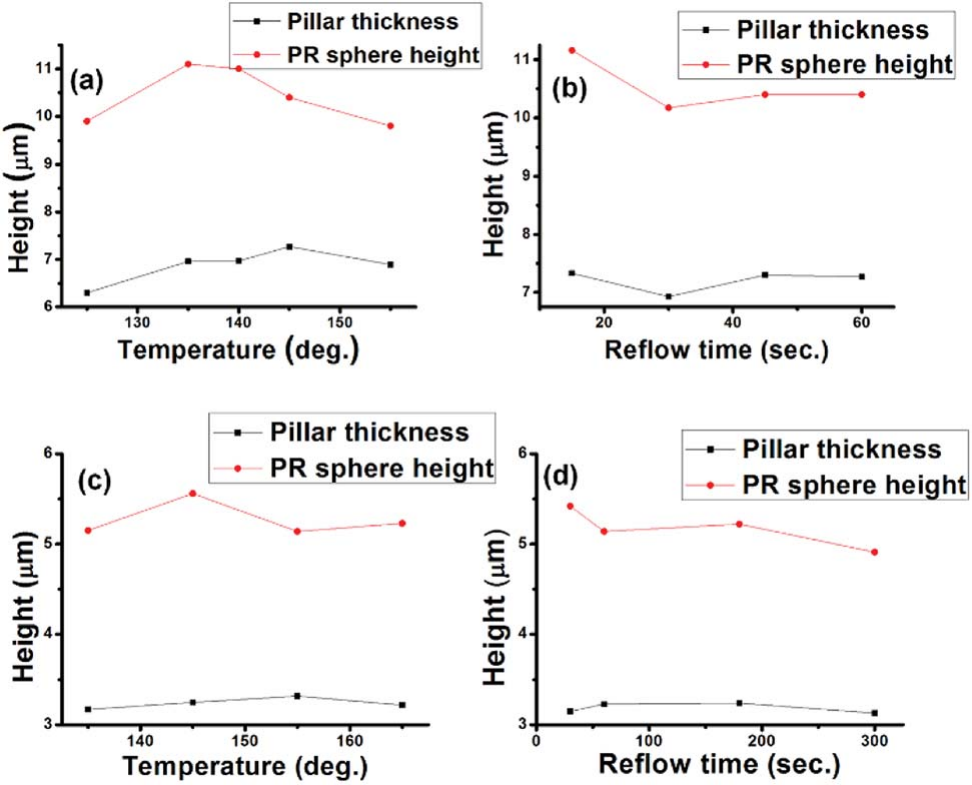
(a and b) Heights of the PR spherical segment *versus* reflow temperature and time with a thick initial pillar thickness; (c and d) heights of the PR spherical segment *versus* reflow temperature and time with a thin initial pillar thickness.

Based on the above results, one typical PR pattern was transferred onto the diamond substrate to form diamond microlenses by an etching process with a selectivity ratio of about 0.1. The selectivity ratio is defined as the diamond etch rate to that of PR in this work. The optical image of the fabricated diamond MLAs indicated a compact arrangement of microlenses. The spherical segment morphologies of these MLAs are shown in Fig. 4(b). The inset of Fig. 4(a) exhibits the cross-sectional profile measured by LCSM, which also indicated a spherical segment shape of the as-fabricated diamond microlens. The focal length of the microlens was calculated to be 552.2 μm based on geometry and optical theory.^17^ Details of the diamond MLAs' interval was investigated with AFM. The roughness of the diamond microlens was evaluated to be 1.5 nm. The average space between two neighbouring microlenses was about 1 μm and the fill factor of the MLAs reached about 90% from that of 86.3% in the fixed contact line case, which is among the highest in diamond MLAs so far. From both Fig. 4(a) and (c), there are etch pits on the surface of the microlens, which are thought to be caused by defects existing between the interface of the photoresist and diamond substrate. These defects may have arisen during the thermal treatment process, which will be studied in our future work.

**Fig. 4:**
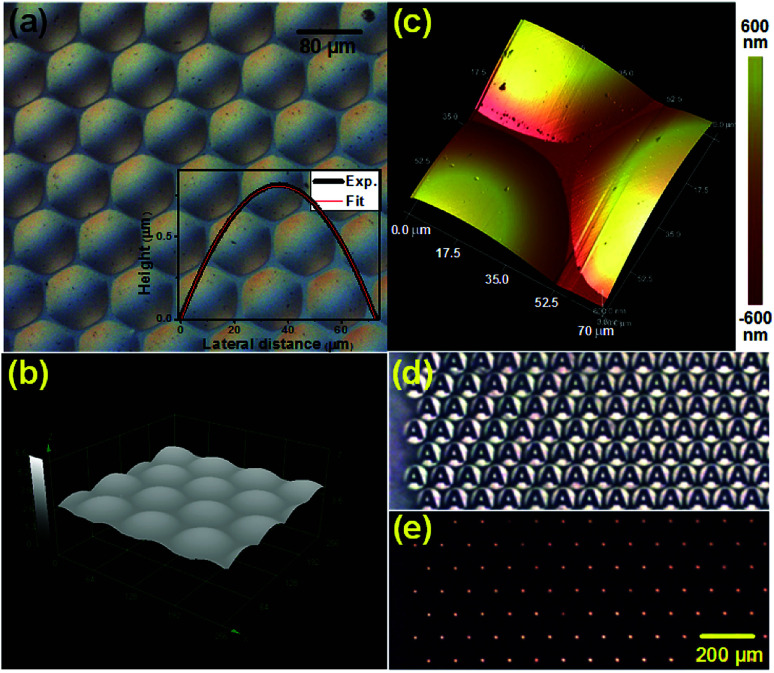
(a) Optical image of as-fabricated diamond MLAs; (b) images of as-fabricated diamond MLAs with laser confocal scanning measurement; (c) AFM image of the gap detail of three neighboring microlenses; (d and e) images projected by the MLAs with “A” and pin-hole photomasks, respectively.

The optical performance of the diamond MLAs was demonstrated with a projection system, which was illustrated and reported on in our previous work.^17^ In the projection experiment, “A” and pin-hole masks were used to test the optical performances, respectively. Fig. 4(d) shows clear images of “A” with a uniform size and shape. As shown in Fig. 4(e), the use of a pin-hole mask leads to arrays of bright spots observed on the false focal plane of the MLAs. The brightness and size of the spot-like images are also uniform.

In our work, the pre-formation of a PR pattern mask is crucial to obtain compact diamond MLAs, since the occupancy ratio of the spherical segment arrays determines that of the diamond MLAs. In the thermal reflow process, PR pillars change from the solid state to fluidic state when the temperature is above its glass transition temperature, then turning to the same spherical shape as the mask. With increasing the thermal reflow time, PR remains in a fluidic state until most of the solvent in PR evaporates out, leading a limited time for contact line enlargement of the PR pattern. In the thin PR case, the solvent in PR is easier to evaporate out from PR due to the lower solvent content and thinner PR thickness in comparison of that in the thick PR case. Therefore, the fixed contact line in the 3.5 μm case could be thought to be due to that the solvent in the pillars evaporates too fast to allow the contact line to fully enlarge. Meanwhile, the height of the final spherical segment is almost independent of the thermal reflow temperature and time. This suggests that the curvature of a microlens with a certain base diameter remains constant with various thermal reflow temperatures or times. Based on our results, a pillar with a thick height is preferred to obtain a high occupancy ratio PR mask with an optimized thermal reflow time. *Via* ICP etching with optimized parameters, PR spherical segment arrays with a high occupancy ratio can be transferred onto a diamond surface. The projection experiment results indicated the uniformity of the structures and the fine imaging property of the as-fabricated MLAs. It also indicated that the MLAs had good consistency regarding their optical performance.^19^ The fine optical properties enable the diamond MLAs to possibly be employed as functional optical devices.

## Conclusions

In summary, compact and uniform diamond MLAs were fabricated by the thermal reflow method with optimized parameters. During the thermal treatment process, the occupancy ratio of PR spherical segments on diamond was found to be dependent on the reflow temperature when the initial PR thickness was thin (3 μm). Also, it was dependent on the reflow temperature and time when the PR thickness was thick (6.5 μm). The difference may be due to the PR fluidity, which was affected by the solvent existing in PR during the thermal process. In addition, the spherical segment height was found to be independent of the reflow temperature and time. Diamond MLAs with a fill factor of about 90% were obtained with ICP etching, and exhibited good optical properties.

## Conflicts of interest

There are no conflicts to declare.

## Supplementary Material
